# Progression-free survival after front line, second line and third line in patients with follicular lymphoma treated in clinical practice

**DOI:** 10.2340/1651-226X.2024.24377

**Published:** 2024-05-06

**Authors:** Aino Rajamäki, Marc Sorigue, Roosa E.I. Prusila, Milla E.L. Kuusisto, Hanne Kuitunen, Esa Jantunen, Santiago Mercadal, Taina Turpeenniemi-Hujanen, Juan-Manuel Sancho, Kaisa Sunela, Outi Kuittinen

**Affiliations:** a Institute of Clinical Medicine, Faculty of Health Sciences, University of Eastern Finland, Kuopio, Finland; b Medical Department, Trialing Health, Barcelona, Spain; c Department of Pediatrics, Kuopio University Hospital, Kuopio, Finland; d Department of Internal Medicine, Länsi-Pohja Central Hospital, Kemi, Finland; e Department of Oncology, Oulu University Hospital, Oulu, Finland; f Department of Medicine, Kuopio University Hospital, Kuopio, Finland; g ICO-Hospital Duran I Reynals, L’Hospitalet, Spain; h Medical Research Center, Oulu University Hospital and Translational Medicine Research Unit, University of Oulu, Oulu, Finland; i Department of Hematology, ICO-Hospital Germans Trias i Pujol, IJC, UAB, Badalona, Barcelona, Spain; j Finnish Medicines Agency FIMEA, Barcelona, Spain; k Department of Oncology, Kuopio University Hospital, Kuopio, Finland

**Keywords:** Follicular lymphoma, survival, progression-free survival, treatment

## Abstract

**Background:**

The modern-day therapeutic landscape for follicular lymphoma (FL) includes a number of highly effective therapies.

**Patients and methods:**

We set out to determine progression-free survival (PFS) after front line, second line, and third line of therapy on the basis of relevant biological characteristics and therapeutic choices. Patients (*n* = 743, 51% females, median 60 years old) diagnosed with grade 1–2 FL between 1997 and 2016 in nine institutions were included.

**Results:**

The median PFS1, PFS2, and PFS3 were 8.1 years (95% confidence interval [CI]: 7–9.3 years), 4.2 years (95% CI: 2.8–5.6 years) and 2.2 years (95% CI 1.7–2.8 years). We found longer PFS1 for (1) females, (2) younger age, (3) lower-risk follicular lymphoma international prognostic index (FLIPI), (4) standard intensity (over low intensity) regimens and (5) immunochemotherapy strategies and (6) maintenance rituximab. We found a shorter PFS2 for patients who received front-line immunochemotherapy. Older age at diagnosis correlated with a shorter PFS3. Intensity of front-line chemotherapy, maintenance, or POD24 status did not correlate with PFS2 or PFS3 in this dataset.

**Interpretation:**

With current immunochemotherapy strategies, the natural course of FL is characterized by shorter-lasting remissions after each relapse. It will be interesting to see whether new therapies can alter this pattern.

## Introduction

Follicular lymphoma (FL) is the most common indolent lymphoma, characterized by translocation t(14;18)(q21;q32) and mutations in epigenetic regulators [1]. It most often presents as asymptomatic lymphadenopathy and in patients with a median age of around 60. The modern-day therapeutic landscape includes a number of highly effective strategies, leading to a median overall survival exceeding 20 years [2, 3]. Therefore, FL must be strategically managed, considering not only short-term efficacy and toxicity but also optimal treatment sequencing, long-term toxicities, quality of life, as well as patients’ preferences and values. We aimed to determine progression-free survival (PFS) after front-line, second-line, and third-line therapy based on relevant biological characteristics of the lymphoma, as well as the choice of front-line therapy, with the goal of further characterizing the expected response to second- and third-line therapy for these patients.

## Patients and methods

Patients diagnosed with grade 1–2 FL between 1997 and 2016 in nine institutions (seven Finnish [including four university hospitals and three central hospitals] and two Spanish university hospitals) were included [4]. Supplementary Figure 1 shows their inclusion by calendar year. Patients were treated based on the clinical guidelines in place at the time [5–7] with the ultimate decision made by agreement between patient and physician, according to standard practice. In-depth characterization of this cohort has been published earlier [4, 8]. All patients with FL were considered for inclusion. The following patients were excluded: (1) those with grade 3 FL. This was done for two reasons. Firstly, because the subtype (3a vs. 3b) was not reported for all patients in our dataset. Secondly, because despite the general idea that grade 1–2 FL and grade 3a FL should be treated similarly, some physicians may approach them differently (i.e. generally favor anthracycline-based front-line and avoid bendamustine- or lenalidomide-based regimens for grade 3a FL [9, 10]). (2) Patients with composite lymphoma at diagnosis and histological transformation before front-line therapy. Those with documented histological transformations during follow-up (*n* = 10, out of 105 patients with a biopsy at the time of relapse; 62/282 (22%) in first relapse, 39/111 (35%) in second relapse, and 4/41 (12%) in third relapse) were not excluded. 3) Patients who underwent watchful waiting and were never actively treated. For those who underwent watchful waiting and were subsequently treated, the first active therapy was considered the first line (and thus included in the PFS1 analysis). This study was conducted according to the declaration of Helsinki, was approved by the review board of the Northern Ostrobothnia Hospital District, and is reported according to the STROBE statement for observational studies [11].

Median and interquartile range and proportions and percentages are given for quantitative and qualitative variables, respectively. For this analysis centering of treatment effectiveness, PFS1 was calculated from the date of front-line treatment start until the date of relapse/progression (detected either due to patient symptoms or on routine scans) or death. PFS2 and PFS3 were calculated from the start of second-line and third-line treatment, respectively, until the time of progression or relapse after those treatment lines or death. For all endpoints, the patients were censored at the time of the last follow-up if relapse, progression, or death had not occurred.

The Kaplan Meyer method was used to draw survival curves, and the log-rank test was used to compare them. Both SPSS and R software (ggplot2, survival, survminer packages) were used for the current study. A formal sample size calculation was not undertaken; rather, all patients diagnosed within the predefined time period and meeting inclusion criteria were included in the study.

## Results

**Figure 1:**
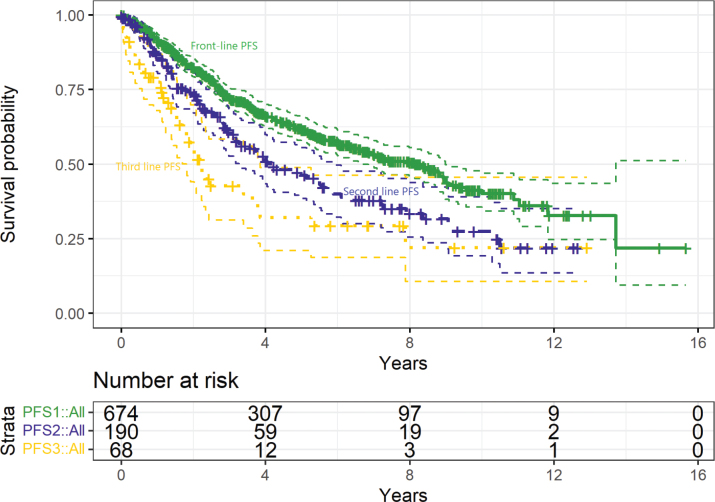
Progression-free survival after front-line (solid line, green, median 8.1 years, 95% CI: 7–9.3 years), second-line (dashed line, purple, median 4.2 years, 95% CI: 2.8–5.6 years), and third-line (dotted line, yellow, median 2.2 years, 95% CI: 1.7–2.8 years) treatment in patients with grade 1–2 FL.

Seven-hundred and forty-three patients were included. The median age was 60, with 51% being female, and 40% had high-risk FLIPI scores (Supplementary Tables 1 and 2). Table 1 details the treatments given, and Figure 1 shows the PFS after front-line, second-line, and third-line treatment, which were a median of 8.1 years (95% confidence interval [CI]: 7–9.3), 4.2 years (2.8–5.6) and 2.2 years (1.7–2.8), respectively.

**Table 1:** Treatment of the patients with grade 1–2 FL included in this study (n = 743).

Characteristic	Front-line	Second-line	Third-line
Patients treated, *n* (%)	697 (94)	231 (31)	72 (10)
Immunochemotherapy			
Any, *n* (%)	493 (71)	130 (56)	44 (61)
Anthracycline-based, *n*	350	47	3
Bendamustine, *n*	60	40	15
Fludarabine-based, *n*	10	10	2
Platinum-based, *n*	-	4	9
Low intensity regimens^a^, *n*	58	22	10
Other^b^, *n*	15	7	5
Chemotherapy without rituximab, *n* (%)			
Any, *n* (%)	69 (10)	52 (23)	16 (22)
Anthracycline-based, *n*	25	10	-
Bendamustine, *n*	1	4	1
Fludarabine-based, *n*	11	17	1
Platinum-based regimen, *n*	-	6	-
Low intensity regimens^a^, *n*	30	12	4
Other^b^, *n*	2	3	10
Rituximab-monotherapy, *n* (%)	28 (4)	14 (6)	4 (6)
Radiation therapy only, *n* (%)	91 (13)	34 (15)	7 (10)
Surgical removal only, *n* (%)	14 (2)	1 (0.4)	-
Stem-cell transplantation consolidation, *n* (%)	44 (6)	31 (13)	7 (10)
Rituximab maintenance (after immunochemotherapy), *n* (%)	207 (42)^c^	47 (36)^c^	11 (25)^c^
Median follow-up, years (95% CI)	6.4 (6.1–6.9)	5.6 (4.9–5.6)	3.4 (2.4–6.3)

a Alkylator-based treatments (cyclophosphamide alone, in combination with prednisone, or with vincristine and prednisone [CVP], chlorambucil) or gemcitabine.

b Other therapies include in front-line: radioimmunotherapy (90Y-ibritumomab tiuxetan), and bortezomib; in second line: MINE/MIME (mesna, ifosfamide, mitoxantrone/methothrexate, and etoposide/mitoguazone, ifosfamide, methotrexate, etoposide), and other heterogeneous chemo-regimen; in third line MINE/MIME, radioimmunotherapy, idealisib, copanlisib, bortezomib, ibrutinib and other heterogeneous chemo-regimen.

c Percentage of patients treated with induction immunochemotherapy.

NB: Front-line treatment information was missing for two patients; CI: confidence interval.

PFS subanalyses can be found in Table 2. Regarding PFS1, we highlight a longer PFS1 for females compared to males, a shorter PFS1 for higher-risk FLIPI categories, a predictable longer PFS1 for standard intensity regimens, for younger age, for immunochemotherapy (over non-immunochemotherapy) and for maintenance rituximab. Regarding PFS2, we find a shorter PFS2 for front-line immunochemotherapy (rather than non-immunochemotherapy) strategies.

**Table 2:** The prognostic impact of patient characteristics, treatment selection and response on PFS-times in different lines of therapy.

Characteristics		*N*	PFS1	*p*	*N*	PFS2	*p*	*N*	PFS3	*p*
**Gender**	Female	333	9.0 (7.2–10.8)	**0.004**	83	4.9 (2.6–7.3)	0.980	28	2.3 (0.7–4.0)	0.662
	Male	328	6.0 (4.7–7.3)		99	4.0 (2.4–5.5)		40	2.1 (1.2–3.0)	
**Age**	<60	329	7.1 (5.5–8.7)	0.043	108	5.1 (3.8–6.4)	0.224	43	3.6 (1.0–6.3)	0.010
	60–69	204	9.0 (6.7–11.3)		43	3.2 (1.4–4.9)		16	2.2 (1.2–3.3)	
	>70	125	6.4 (5.0–7.8)		31	2.8 (0.5–5.0)		9	0.6 (0.1–1.0)	
**FLIPI score**	0–1	209	NR (5-year 65% [69–73])	**0.002**	53	5.1 (3.0–7.3)	0.107	19	2.3 (0.7–4.0)	0.678
	2	174	8.8		37	3.2 (2.5–3.8)		16	3.8 (0.3–7.3)	
	3–5	214	6.7 (4.5–8.8)		62	3.1 (2.5–3.8)		23	1.7 (0.6–2.1)	
**Front-line induction backbone (Sup. Figure 2)**	Anthracycline	363	8.8 (7.5–10.1)	**<0.001**	82	4.0 (2.9–5.1)	0.495	34	2.0 (1.2–2.8)	0.985
	Bendamustine/fludarabine	80	NR (5-year 72% [61–85])		10	3.9 (0–10.0)		4	0.6	
	Low intensity regimens^a^	111	4.2 (3.0–5.5)		48	3.2 (1.8–4.6)		22	2.2 (1.8–2.7)	
**Front-line induction strategy (Sup. Figure 3)**	Immunochemot.	480	8.8 (7.4–10.2)	**<0.001**	101	3.1 (2.3–3.9)	**0.009**	41	2.0 (1.5–2.5)	0.581
	No immunochemot.	182	5.3 (3.4–7.3)		81	6.1 (3.3–9.0)		27	3.4 (1.0–5.8)	
**Rituximab maintenance after front-line ICT**	Yes	193	NR (5-year 74% [67–82])	**<0.001**	28	3.0 (2.7–3.2)	0.589	9	NR (5-year 59% [32–100])	0.247
	No	287	7.1 (5.1–9.1)		73	3.5 (2.2–4.7)		32	1.9 (1.6–2.2)	
**Front-line response duration**	POD24	111	NA	NA	66^b^	3.3 (0.9–5.7)	0.463	29^b^	2.2 (0.5–3.9)	0.581
	non-POD24	486	NA		113^b^	4.8 (3.5–6.2)		37^b^	2.2 (1.2–3.3)	

PFS: Progression-free survival; NR: not reached; FLIPI: Follicular Lymphoma International Prognostic Index; ICT: immunochemotherapy; POD24: progression of disease within 24 months of front-line therapy; NA: Not applicable.

Data are median (95% confidence interval) and reported in years. Only patients who received treatment are included in these analyses.

a Alkylator-based regimens (CVP, RCVP), alkylator monotherapy (cyclophosphamide or chlorambucil) or rituximab monotherapy. Within group survival estimates are in Supplementary Table 2.

b Second-line treatments for non-POD24 patients included: bendamustine-based (*n* = 34/113, 30%), anthracycline-based (*n* = 25, 22%), alkylator-based (*n* = 12, 11%), fludarabine-based (*n* = 1, 1%), or platinum-based therapy (*n* = 1, 1%), rituximab monotherapy (*n* = 19, 17%), radiotherapy (*n* = 20, 18%), or other (*n* = 1, 1%). For POD24 patients they included: bendamustine-based (*n* = 9/66, 14%), anthracycline-based (*n* = 14, 21%), alkylator-based (*n* = 7, 11%), fludarabine-based (*n* = 11, 17%), or platinum-based therapy (*n* = 7, 11%), rituximab monotherapy (*n* = 5, 8%), radiotherapy (*n* = 9, 14%), or other (*n* = 4, 6%). Stem cell transplant consolidation after second-line induction in 21/113 (19%) patients in the non-POD24 group and 10/66 (15%) in the POD24 group.

NB: Bold values indicate statistical significance

## Discussion

In this study of patients with grade 1–2 FL, we: (1) confirmed decreasing PFS after each relapse, (2) confirmed different PFS1 associated with a number of biological and treatment-related variables, (3) found a longer PFS2 for patients treated in the front-line without immunochemotherapy compared to those treated with immunochemotherapy and (4) found either no association or a loose association between PFS2 or PFS3 and biological and treatment-related variables and response to the front line.

We found that PFS in FL decreases after each line of therapy, in line with previous data [12–15]. This is a pattern globally observed in all malignancies and appears to be secondary to clonal selection, as malignant cells are exposed to anticancer agents [16].

Overall, PFS seems satisfactory in the early lines of therapy and translates into prolonged overall survival (OS) – 72.4% at 10 years in this same cohort [4] – but drops substantially particularly after the second line. Indeed, PFS3 is barely over 2 years, despite frequent use of immunochemotherapy, highlighting the need for new strategies. Fortunately, strategies that were not available at the time of this study – patients were diagnosed in 2016 at the latest, to ensure a long enough follow-up – are currently available, including rituximab-lenalidomide, anti-CD3xCD20 bispecific antibodies, or CAR T-cell therapy [2].

We also report several correlations between PFS1 and patient variables (gender and age), disease-related variables (FLIPI score), treatment selection, and length of remission (POD24). The adverse impact on PFS1 of high-risk FLIPI scores, age, use of low-intensity or non-immunotherapy-containing regimens, a no-maintenance strategy, or POD24 are consistent in published data [17]. The correlation between sex and outcome is more controversial and inconsistent [18–23]. It is possible that male sex has a small negative association with prognosis that reaches significance only in some, but not all, studies, consistent with what has been documented in diffuse large B-cell lymphoma [24, 25].

Perhaps the most novel and interesting datapoints of this analysis are the associations between baseline patient-, disease-, or treatment-related variables and PFS2 and PFS3. Most baseline variables or responses to front-line treatment did not have a strong correlation with PFS2 or PFS3. The one exception is that using non-immunochemotherapy strategies in the front line was associated with a notably longer PFS2 than using immunochemotherapy strategies in the front line. This fits well within the framework of the clonal selection model [16], as these patients can receive in the second-line therapeutic agent/s of a class that they have not previously been exposed to – be it immunotherapy, be it chemotherapy – , unlike those treated in the front line with immunochemotherapy. These results seem to indicate that a shorter PFS1 in the front line can be offset by subsequent lines of therapy, partly challenging the extended notion that obtaining the longer possible front-line PFS1 is essential [26, 27]. This notion is also supported by the long-term survival evidence from patients treated with rituximab monotherapy in the front line [28] or with a no-maintenance approach, which does not impact OS negatively despite notably inferior PFS1 compared to those who receive maintenance [29] and the lack of correlation between PFS1 and OS in FL [30, 31].

Another interesting finding, albeit a negative one, is the lack of an association between PFS2 and POD24 although an adverse impact on PFS2 of small magnitude cannot be ruled out. POD24 is associated with a poor OS in patients treated with immunochemotherapy, a finding that was first described in the mid-00s [32] and that has been replicated [33], including with this very dataset [34]. However, since its first description, other analyses have provided substantial nuance to this finding; replication studies have shown that the prognostic impact is of a smaller magnitude than initially reported, particularly in PET-staged patients [35] and that the poorest OS is likely restricted to primary refractory or very early relapsing (<6 or < 12 months) patients or to those with histological transformation [35–37]. The lack of association between POD24 and PFS2 in our study seems to indicate that POD24 is not always an ominous prognostic sign and that a substantial number of patients with POD24 can be rescued with subsequent lines of therapy, as suggested by a previous analysis [38]. However, patients with POD24, particularly primary refractory with an aggressive clinical course could have died before receiving the second line, and this would not be captured by second-line survival estimates. While this is an uncommon occurrence, it is a catastrophic one that should not be forgotten. Then, patients with POD24 may also be treated more aggressively in the second line, which might partially offset a potential worse prognosis of these patients. While not the object of the present analysis, we did assess whether this might have been the case – to the extent possible, as we could not assess cumulative drug exposure or dose intensity – and we found some differences in the use of rituximab monotherapy (greater in non-POD24) and platinum-based therapy (greater in POD24 patients). However, the differences appeared rather subtle and unlikely to drive the relatively small survival differences between the groups. Of interest, we found no sign of a difference in the use of stem cell transplant between POD24 and non-POD24 patients, as noted in previous cohorts [14].

Regarding PFS3, the analysis is limited by shorter follow-up and a limited number of patients, but the baseline differences we could analyze here have little association with PFS3, likely because of intervening events and treatments.

A number of limitations should be acknowledged. The most important one is the observational – and retrospective – nature of this analysis. Several biases, including indication bias, apply and prevent drawing conclusions about the causal link between treatment decisions and PFS estimates. Only 10 patients had documented histological transformation during follow-up. We lacked detailed data on the histological findings and did not have complete data on potential transformations that were not histologically confirmed. While this is a study shortcoming, the small number of transformations, which aligns with the decreasing number of such events in patients treated with immunochemotherapy and staged by PET versus in earlier series [35, 39, 40], as well as with the lower risk of transformation of grade 1–2 FL (compared to grade 3 [40, 41]), suggests that this would not have a major impact on the survival estimates provided here. The inclusion of only grade 1–2 FL should also be kept in mind when evaluating the treatment patterns and survival estimates reported. Despite aiming to make the study representative of patients with grade 1–2 FL, a broad comparison with other studies with different designs [33, 42, 43] shows minor potential differences in selected data points, such as median age and percentage of patients treated with R-single, indicating that elderly patients and those with very indolent disease might be somewhat under-represented in our cohort. Finally, the exploratory nature of the study and the multiple tests ran, raise concerns about false-positive findings. However, most of our results seem to align with previous data and inferences from other analyses, which lowers the likelihood of them being false positives and supports our conclusions. A few findings do not clearly align with previous studies, and these should be more carefully considered. Other limitations include the short follow-up, particularly for PFS2 and PFS3, as well as the small number of patients for the PFS3 analyses, which prevent us from excluding differences of small or, in some instances, moderate magnitude. As a strength of the study, we note the precise estimates that follow a large dataset and the reliability of the results of an unselected patient cohort.

To conclude, in this retrospective analysis of grade 1–2 FL, we confirm a decrease in PFS after each relapse, and we find longer PFS2 for patients treated in the front line without immunochemotherapy but find few other associations between PFS2 or PFS3 and baseline patient- and disease-related variables, front-line treatment strategy, or response.

## Data Availability

For legal reasons, individual-level patient data cannot be made available. Access is only allowed to authorized researchers after approval by the appropriate national body.
